# Model for the diffuse reflectance in spatial frequency domain imaging

**DOI:** 10.1117/1.JBO.28.4.046002

**Published:** 2023-04-07

**Authors:** Anouk L. Post, Dirk J. Faber, Ton G. van Leeuwen

**Affiliations:** a The Netherlands Cancer Institute, Department of Surgery, Amsterdam, The Netherlands; b Amsterdam University Medical Centers, Department of Biomedical Engineering and Physics, Amsterdam, The Netherlands

**Keywords:** spatial frequency domain imaging, structured light imaging, diffuse reflectance, partial current boundary condition, extended boundary condition

## Abstract

**Significance:**

In spatial frequency domain imaging (SDFI), tissue is illuminated with sinusoidal intensity patterns at different spatial frequencies. For low spatial frequencies, the reflectance is diffuse and a model derived by Cuccia et al. (doi 10.1117/1.3088140) is commonly used to extract optical properties. An improved model resulting in more accurate optical property extraction could lead to improved diagnostic algorithms.

**Aim:**

To develop a model that improves optical property extraction for the diffuse reflectance in SFDI compared to the model of Cuccia et al.

**Approach:**

We derive two analytical models for the diffuse reflectance, starting from the theoretical radial reflectance R(ρ) for a pencil-beam illumination under the partial current boundary condition (PCBC) and the extended boundary condition (EBC). We compare both models and the model of Cuccia et al. to Monte Carlo simulations.

**Results:**

The model based on the PCBC resulted in the lowest errors, improving median relative errors compared to the model of Cuccia et al. by 45% for the reflectance, 10% for the reduced scattering coefficient and 64% for the absorption coefficient.

**Conclusions:**

For the diffuse reflectance in SFDI, the model based on the PCBC provides more accurate results than the currently used model by Cuccia et al.

## 1 Introduction

Spatial frequency domain imaging (SFDI, also known as modulated imaging or structured light imaging) is a technique that facilitates wide-field imaging of tissue optical properties^1^^,^^2^ and has gained considerable interest in recent years for a range of applications,^3^ such as tumor margin assessment in breast cancer^4^ and assessment of diabetic feet.^5^ In SFDI, tissue is illuminated with sinusoidal intensity patterns and the reflected intensity is captured by a camera. Scattering and absorption of light by the tissue change the amplitude of the reflected intensity pattern (but not its spatial frequency). The amplitude of the reflected intensity patterns, $M_{\mathrm{AC}}$, depends on the tissue optical properties, the projected spatial frequency, and the properties of the optical system itself. By measuring the reflectance at two or more spatial frequencies, the tissue optical properties can be extracted from SFDI measurements. Compared to other wide-field optical techniques, such as conventional multi- and hyperspectral imaging, SFDI has the advantage that it can be performed using only a few wavelengths, improving acquisition time. Furthermore, by changing the projected spatial frequency, the interrogation depth of SFDI can be modified^6^ to match the clinical application.

To extract optical properties from SFDI measurements, the reflected intensity is first demodulated. The amplitude modulation $M_{\mathrm{AC}}$ for any tissue location x, and projected spatial frequency $f_{x}$ can be expressed as

$$M_{\mathrm{AC}}(x,f_{x})=I_{0}(x,f_{x})\cdot {\mathrm{MTF}}_{\mathrm{SYS}}(x,f_{x})\cdot R_{\text{tissue}}(x,f_{x},\mu_{a},\mu_{s},p(\theta )),$$ (1)

where $I_{0}$ is the projected intensity pattern, ${\mathrm{MTF}}_{\mathrm{SYS}}$ is the modulation transfer function of the system, and $R_{\text{tissue}}$ is the reflectance from the tissue which also depends on the tissue optical properties—the absorption coefficient $\mu_{a}$, the scattering coefficient $\mu_{s}$, and the phase function p(θ). The phase function describes the probability of scattering at a certain angle θ with respect to its previous direction. In general, two methods exist to demodulate the reflectance. For a detailed description, we refer to.^3^ In the single-pixel demodulation method, three images are acquired with the same projected spatial frequency, but phase-shifted 2π/3. From these three images, $M_{\mathrm{AC}}$ is calculated through a simple analytical function. In the multipixel demodulation method, a single image is acquired and $M_{\mathrm{AC}}$ is calculated by taking a Fourier transform of a line or the entire image.

To obtain $R_{\text{tissue}}$ from $M_{\mathrm{AC}}$, the modulation transfer function of the optical system has to be determined. ${\mathrm{MTF}}_{\mathrm{SYS}}$ is obtained through a calibration step where the $M_{\mathrm{AC}}$ of a reference sample is measured for which the optical expected reflectance $R_{\mathrm{ref}}$ is known,

$${\mathrm{MTF}}_{\mathrm{SYS}}(x,f_{x})=\frac{M_{\mathrm{AC},\mathrm{ref}}}{I_{0}(x,f_{x})\cdot R_{\mathrm{ref}}}.$$ (2)

To extract tissue optical properties from SFDI measurements of $R_{\text{tissue}}$, two main approaches exist. The first approach is to use an analytical model based on physics that relates $R_{\text{tissue}}$ to the tissue optical properties.^2^^,^^7^ The second approach is to use Monte Carlo (MC) simulations to relate $R_{\text{tissue}}$ to tissue optical properties, either through a look-up-table approach or based on machine learning.^8^^–^^10^ In this manuscript, we will focus on the first approach, an analytical model based on physics.

Two analytical models exist for SFDI, the model of Cuccia et al. for the diffuse regime^2^ and the model of Kanick et al. for the subdiffuse regime.^7^ The model of Kanick et al. is a semiempirical model based on MC simulations and the model of Cuccia et al. was derived from diffusion theory. Whether measurements are in the diffuse or subdiffuse regime depends on the projected spatial frequency and tissue optical properties. In this manuscript, we will focus on modeling the diffuse reflectance in SFDI. Diffusion theory is generally thought to be valid for $f\ll \mu_{\mathrm{tr}}$ and $\mu_{s}^{\prime }\gg \mu_{a}$ (where $\mu_{\mathrm{tr}}=\mu_{a}+\mu_{s}^{\prime }$). Under the assumption of linearity of the medium (i.e., the frequency and phase of the modulated incident light are maintained in the modulated fluence rate in the tissue), Cuccia et al. obtained the following diffusion equation:^2^

$$\nabla_{z}^{2}\varphi (z)-\mu_{\mathrm{eff}}^{\prime 2}\varphi (z)=-3\mu_{\mathrm{tr}}S(z),$$ (3)

where φ(z) is the fluence rate at depth z, $\mu_{\mathrm{eff}}^{\prime }=\sqrt{\left(\mu_{\mathrm{eff}}^{2}+k^{2}\right)}$ is the effective attenuation coefficient that takes into account the influence of the projected spatial frequency $f=\frac{k}{2\pi }$, $\mu_{\mathrm{eff}}=\sqrt{\mu_{a}/D}$, and D is the diffusion coefficient $D=1/(3(\mu_{s}^{\prime }+\mu_{a}))$, S(z) is a source term, and $\mu_{\mathrm{tr}}$ is the transport coefficient. The only difference with the diffusion equation for a uniform plane illumination (k=0) is in the scalar parameter $\mu_{\mathrm{eff}}^{\prime }$. Therefore, any approach to obtain the reflectance for a uniform plane illumination can be used for SFDI by replacing $\mu_{\mathrm{eff}}$ with $\mu_{\mathrm{eff}}^{\prime }$. For the diffuse reflectance for a uniform plane illumination, Cuccia et al. used the model of Svaasand et al.^11^ for the diffuse reflectance for an infinitely wide illumination source and obtained the following model:^2^

$$R_{\text{Cuccia}}(k)=\frac{3a^{\prime }}{\left(2{{A\mu }_{\mathrm{eff}}}^{\prime }/\mu_{\mathrm{tr}}+3)(\mu_{\mathrm{eff}}\prime /\mu_{\mathrm{tr}}+1\right)},$$ (4)

where $a^{\prime }=\mu_{s}^{\prime }/\mu_{\mathrm{tr}}$ is the reduced albedo and $A=(1+R_{\mathrm{eff}})/(1-R_{\mathrm{eff}})$ describes the influence of the refractive index mismatch between the sample and the surrounding medium. Please note that a different expression for A was used by Cuccia et al. ($A_{\text{Cuccia}}=[1-R_{\mathrm{eff}}]/[2(1+R_{\mathrm{eff}})]$ and therefore Eq. (4) might seem different from their manuscript but the equation is only rewritten. In this paper, we propose a new model for the diffuse reflectance in SFDI and show that it reduces the median error in the estimated reflectance and extracted optical properties.

## 2 Modeling the Diffuse Reflectance in SFDI

As an alternative to directly solving the diffusion equation for a uniform plane illumination, a tissue “impulse response” can be obtained by computing the reflectance as a function of radial distance, R(ρ), for illumination by an infinitely narrow pencil beam. The reflectance as a function of spatial frequency is then found by a 2D Fourier transform, which in the case of cylindrical symmetry is the same as computing the zeroth order Hankel transform:

$$R(k)=2\pi \int \rho \cdot J_{0}(k\rho )\cdot R(\rho )d\rho ,$$ (5)

where $J_{0}$ is the zeroth order Bessel function of the first kind. This approach is often used to translate MC simulations that generate R(ρ) to reflectance values $R_{\text{tissue}}(k)$ for SFDI for a range of spatial frequencies.^2^^,^^7^ The same principle can equally well be applied to analytical solutions of R(ρ).

In general, diffusion theory assumes infinite media. For semi-infinite media such as tissue, the refractive index mismatch between the sample and the medium above it results in a significant fraction of radiant energy being reflected back into the sample upon interaction with the boundary. Analytical expressions for R(ρ) can be obtained by imposing appropriate boundary conditions at the interface between the sample and the (non-scattering) medium above it. Analytical expressions for R(ρ) in the diffuse regime are available for different (yet equivalent) boundary conditions, i.e., the partial current boundary condition (PCBC) as proposed by Keijzer et al.^12^ and the extended boundary condition (EBC) as proposed by Farrell et al.^13^ For the PCBC, the irradiance at the boundary is set equal to the integral of the reflected radiance^12^ and for the EBC, the fluence rate is set to zero at an extended boundary located at a distance $z_{b}$ outside the sample. There is no theoretical reason to prefer the PCBC over the EBC, the difference merely demonstrates a limitation of diffusion theory.^14^ The two boundary conditions result in the following expressions for the reflectance:

$$R_{\mathrm{PCBC}}(\rho ,z_{0})=\frac{1}{4\pi 2\mathrm{AD}}[\frac{\exp(-\mu_{\mathrm{eff}}\cdot r_{1})}{r_{1}}-\frac{\exp(-\mu_{\mathrm{eff}}\cdot r_{2})}{r_{2}}],$$ (6)

and

$$R_{\mathrm{EBC}}(\rho ,z_{0})=\frac{1}{4\pi }[z_{0}(\mu_{\mathrm{eff}}+\frac{1}{r_{1}})\cdot \frac{e^{-\mu_{\mathrm{eff}}\cdot r_{1}}}{r_{1}^{2}}+(z_{0}+2z_{b})(\mu_{\mathrm{eff}}+\frac{1}{r_{2}})\cdot \frac{e^{-\mu_{\mathrm{eff}}\cdot r_{2}}}{r_{2}^{2}}],$$ (7)

where $\mu_{\mathrm{eff}}=\sqrt{\mu_{a}/D}$, $r_{1}=\sqrt{z_{0}^{2}+\rho^{2}}$, and $r_{2}=\sqrt{(z_{0}+2z_{b}{)}^{2}+\rho^{2}}$, $z_{b}=2\mathrm{AD}$. D is the diffusion coefficient $D=1/(3(\mu_{s}^{\prime }+\mu_{a}))$. A describes the influence of the mismatch between the refractive index of the sample, $n_{i}$, and the surrounding medium, $n_{e}$, which is equal to^15^^,^^16^

$$A=\frac{1+3\int_{0}^{\frac{\pi }{2}}R_{F}(\cos\;\theta )\cos^{2}\;\theta \;\sin\;\theta d\theta }{1-2\int_{0}^{\frac{\pi }{2}}R_{F}(\cos\;\theta )\cos\;\theta \;\sin\;\theta d\theta },$$ (8)

where

$$R_{F}=\{\begin{array}{ll} \frac{1}{2}{\left(\frac{n_{i}\;\cos\;\theta -n_{e}\;\cos\;\theta_{r}}{n_{i}\;\cos\;\theta +n_{e}\;\cos\;\theta_{r}}\right)}^{2}+\frac{1}{2}(\frac{n_{e}\;\cos\;\theta -n_{i}\;\cos\;\theta_{r}}{n_{e}\;\cos\;\theta +n_{i}\;\cos\;\theta_{r}}{)}^{2} & \text{for }0<\theta <\theta_{c}\\ 1 & \text{for }\theta \geq \theta_{c}=\arcsin(\frac{n_{e}}{n_{i}}) \end{array},$$ (9)

where $\theta_{r}$ is the angle of refraction.

The reflectance as a function of radial distance is then found as $R(\rho )=\int_{z_{0}=0}^{\infty }R(\rho ,z_{0})S(z_{0}){\mathrm{dz}}_{0}$, where S is the source term, for which we use a distributed source term $S(z)=a^{\prime }\mu_{\mathrm{tr}}\;\exp(-\mu_{\mathrm{tr}}z)$. To obtain our new models for the SFDI reflectance we integrate R(ρ) for each boundary condition over the radial coordinate ρ from 0 to ∞ and we replace $\mu_{\mathrm{eff}}$ with $\mu_{\mathrm{eff}}^{\prime }$.

Performing the integrations for $R_{\mathrm{PCBC}}(\rho ,z_{0})$ and $R_{\mathrm{EBC}}(\rho ,z_{0})$ over $z_{0}$ and ρ we arrive at

$$R_{\mathrm{PCBC}}(k)=\frac{a^{\prime }}{\frac{4A}{3}{\mu_{\mathrm{eff}}}^{\prime }/\mu_{\mathrm{tr}}}\frac{\left[1-\exp(-\frac{4A}{3}{\mu_{\mathrm{eff}}}^{\prime }/\mu_{\mathrm{tr}})\right]}{1+{\mu_{\mathrm{eff}}}^{\prime }/\mu_{\mathrm{tr}}},$$ (10)

and

$$R_{\mathrm{EBC}}(k)=\frac{a^{\prime }}{2}[1+\exp(-\frac{4A}{3}{\mu_{\mathrm{eff}}}^{\prime }/\mu_{\mathrm{tr}})]\frac{1}{\left[{\mu_{\mathrm{eff}}}^{\prime }/\mu_{\mathrm{tr}}+1\right]}.$$ (11)

## 3 Methods

Since the model of Cuccia et al. was based on the PCBC, we first compare $R_{\text{Cuccia}}(k)$ and $R_{\mathrm{PCBC}}(k)$ to the Hankel Transform of $R_{\mathrm{PCBC}}(\rho ,z_{0})$. To calculate this Hankel Transform with Matlab we first integrated $R_{\mathrm{PCBC}}(\rho ,z_{0})$ over $z_{0}$ from 0 to 100 mm and r from 0 to 500 mm (increasing the integration limits did not change the results). Next, to compare the accuracy of the three different models ($R_{\text{Cuccia}}(k)$, $R_{\mathrm{PCBC}}(k)$ and $R_{\mathrm{EBC}}(k)$) we performed MC simulations to obtain the reflectance versus radial distance, $R_{\mathrm{MC}}(\rho )$. To calculate the reflectance measured by SFDI, $R_{\mathrm{MC}}(k)$, we performed a Hankel Transform. For each model, we calculated the relative error (RE) in the reflectance with respect to $R_{\mathrm{MC}}(k)$ for all the simulations

$$\mathrm{RE}=\frac{\left|R_{\text{model}}-R_{\mathrm{MC}}\right|}{R_{\mathrm{MC}}}.$$ (12)

We also determined the relative errors in the values of $\mu_{a}$ and ${\mu_{s}}^{\prime }$ that would be obtained for each of the models if they would be used to fit measured reflectance values obtained with spatial frequencies of 0 and $0.5\;{\mathrm{mm}}^{-1}$.

### 3.1 Monte Carlo simulations

We simulated a pencil beam illumination and collected photons versus radial distance from the source with a 0.001-mm bin size and $4\cdot 10^{5}$ bins (regardless of their angle upon detection) and we performed a Hankel Transform to obtain the reflectance for a given spatial frequency. We simulated tissues with all combinations of $\mu_{a}=[0.001,0.005,0.01,0.05,0.1]\;{\mathrm{mm}}^{-1}$, $\mu_{s}^{\prime }=[1,5,10,20,50]\;{\mathrm{mm}}^{-1}$, and two different phase functions. One set of simulations was done with a Henyey-Greenstein (HG) phase function with $g_{1}=0.9$, and a second set of simulations was done with a two-term HG (TTHG) phase function since the majority of published phase function measurements are best described by a TTHG.^17^ We used a TTHG phase functionwith a scattering anisotropy $g_{1}$ of ∼0.83, using the following parameters: $p(\theta )=0.45\cdot P_{\mathrm{HG}}(g_{\mathrm{HG}}=0.95)+0.05\cdot p_{\mathrm{HG}}(g_{\mathrm{HG}}=-0.2)$, where $p_{\mathrm{HG}}$ denotes a regular HG phase function. For each set of optical properties, we used spatial frequencies such that $\mu_{s}^{\prime }f^{-1}$ ranged from 0.1 to 1000 with 20 equal steps on a log-scale for each value of ${\mu_{s}}^{\prime }$. We simulated a refractive index of the tissue of 1.33 and the medium above it of 1.00 (A=2.515). For diffusion theory to hold, $f\ll {\mu_{\mathrm{tr}}}^{\prime }$, so we excluded simulations where $f>\frac{1}{5}\cdot {\mu_{\mathrm{tr}}}^{\prime }$. We performed each simulation three times and ensured we launched enough photons so that the standard deviation over the mean reflectance was <1%. To determine the accuracy of the models we compared these to the reflectance values averaged over these three simulations.

## 4 Results

**Fig. 1:**
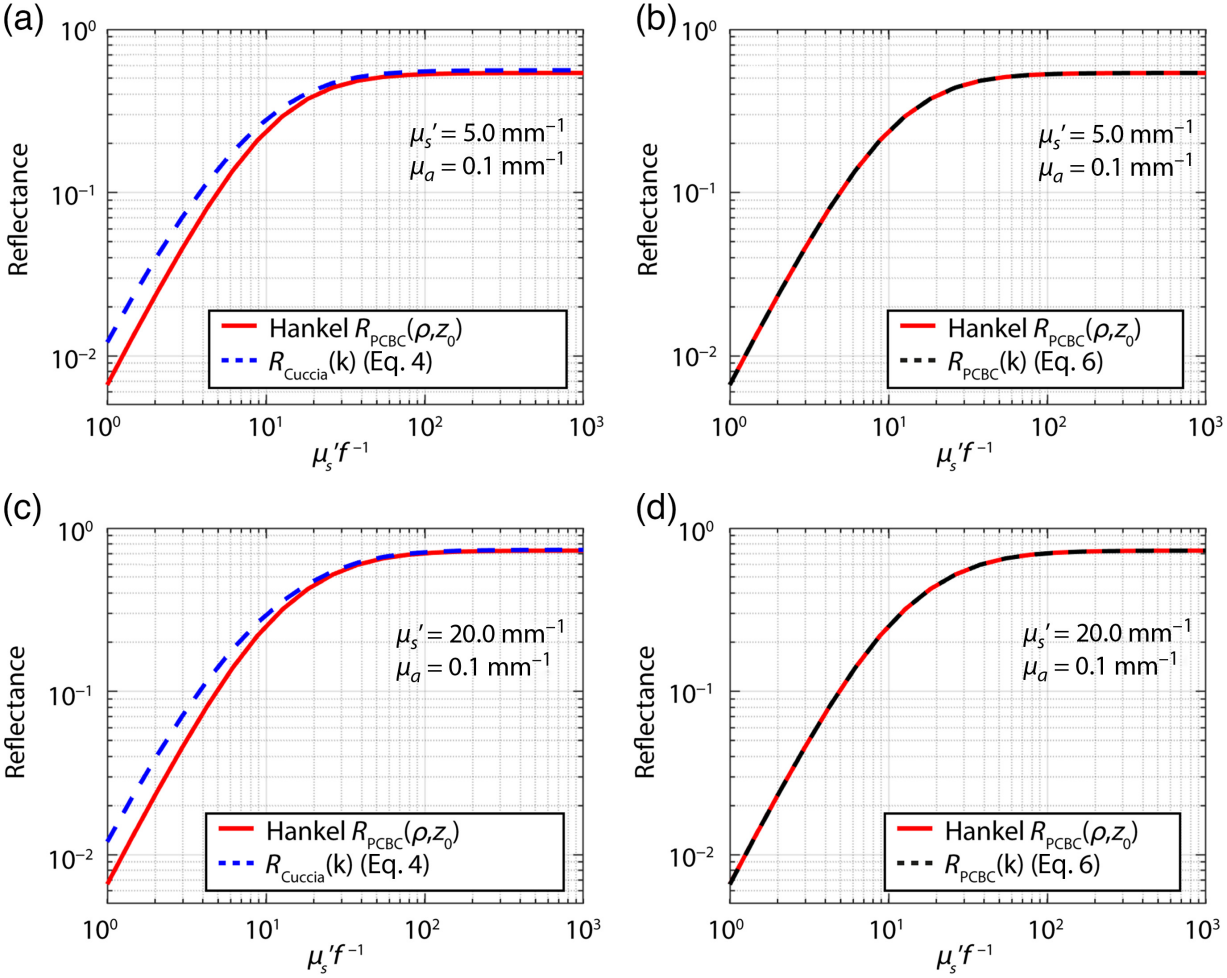
(a) and (c) Reflectance versus $\mu_{s}^{\prime }f^{-1}$ calculated with the Hankel transform of Eq. (6) for the $R_{\mathrm{PCBC}}(\rho ,z_{0})$ (red lines) compared to $R_{\text{Cuccia}}$ [Eq. (4), blue lines] and (b) and (d) the model for $R_{\mathrm{PCBC}}(k)$ [Eq. (10), black lines] for two sets of optical properties as indicated in each subfigure. $R_{\text{Cuccia}}$ does not match the Hankel transform of Eq. (6) for the $R_{\mathrm{PCBC}}(\rho ,z_{0})$, while $R_{\mathrm{PCBC}}(k)$ does match.

We first compared $R_{\text{Cuccia}}(k)$ [Eq. (4)] and $R_{\mathrm{PCBC}}(k)$ [Eq. (10)] to the Hankel Transform [Eq. (5)] of $R_{\mathrm{PCBC}}(\rho ,z_{0})$ [Eq. (6)], since they are both based on the PCBC (Fig. 1). While the model of Cuccia et al. is based on the PCBC, it does not overlap with the Hankel Transform of $R_{\mathrm{PCBC}}(\rho ,z_{0})$. The derived model $R_{\mathrm{PCBC}}(k)$ does overlap with the Hankel Transform of $R_{\mathrm{PCBC}}(\rho ,z_{0})$.

**Fig. 2:**
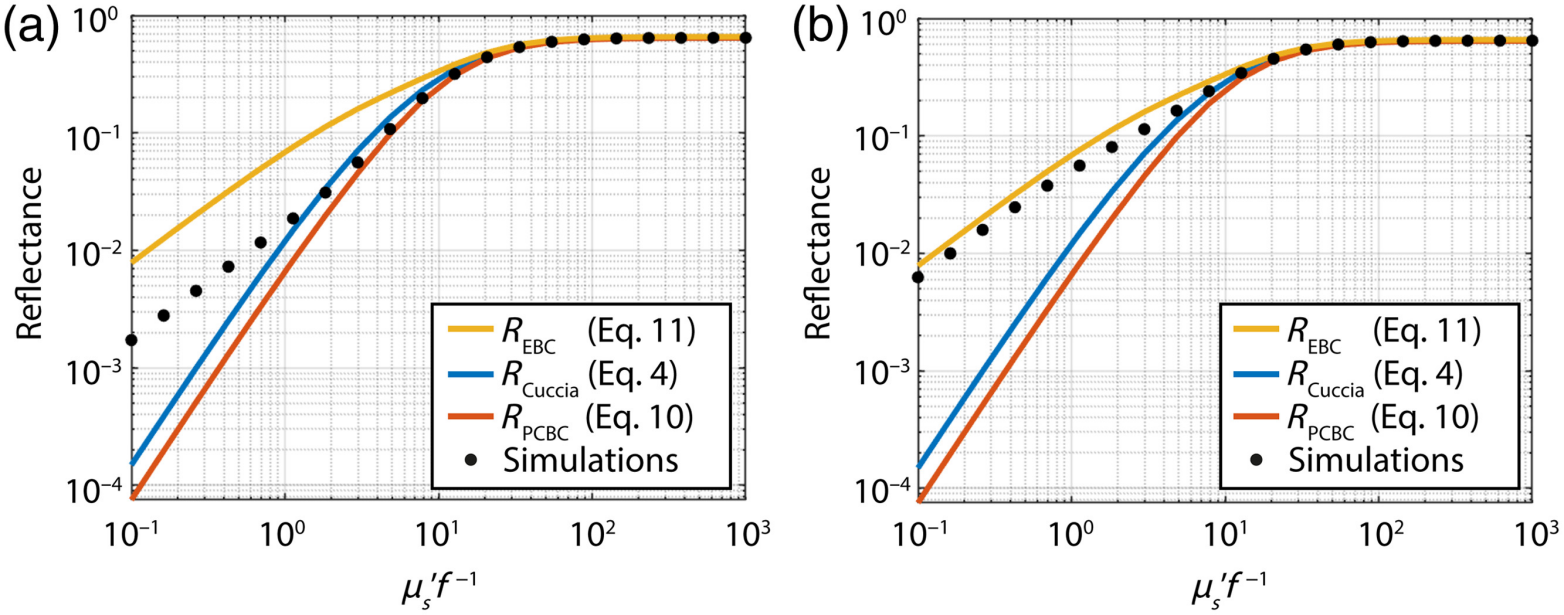
(a) Models for the diffuse reflectance compared to the total simulated reflectance for $\mu_{a}=0.01\;{\mathrm{mm}}^{-1}$ and $\mu_{s}^{\prime }=1\;{\mathrm{mm}}^{-1}$ for the HG phase function with $g_{1}=0.9$ and (b) the two-term HG phase function with $g_{1}=0.83$. In both, the diffuse (higher values of $\mu_{s}^{\prime }f^{-1}$) and the subdiffuse regime (lower values of $\mu_{s}^{\prime }f^{-1}$) the diffuse reflectance should be equal to or lower than the total reflectance. This is only true for $R_{\mathrm{PCBC}}$ in this figure. Thus, $R_{\text{Cuccia}}$ and $R_{\mathrm{EBC}}$ overestimate the diffuse reflectance.

An example of the reflectance versus $\mu_{s}^{\prime }f^{-1}$ for the different models and MC simulations is shown in Fig. 2 for $\mu_{a}=0.01\;{\mathrm{mm}}^{-1}$ and $\mu_{s}^{\prime }=5\;{\mathrm{mm}}^{-1}$. For higher spatial frequencies, measurements are in the subdiffuse regime, where the total reflectance should be equal to the sum of the semiballistic and diffuse reflectance.^18^ Thus, for any value of $\mu_{s}^{\prime }f^{-1}$ the diffuse reflectance should be equal to or smaller than the total reflectance. In Fig. 2, this is only true for $R_{\mathrm{PCBC}}$, which indicates that both $R_{\text{Cuccia}}$ and $R_{\mathrm{EBC}}$ overestimate the diffuse reflectance.

**Fig. 3:**
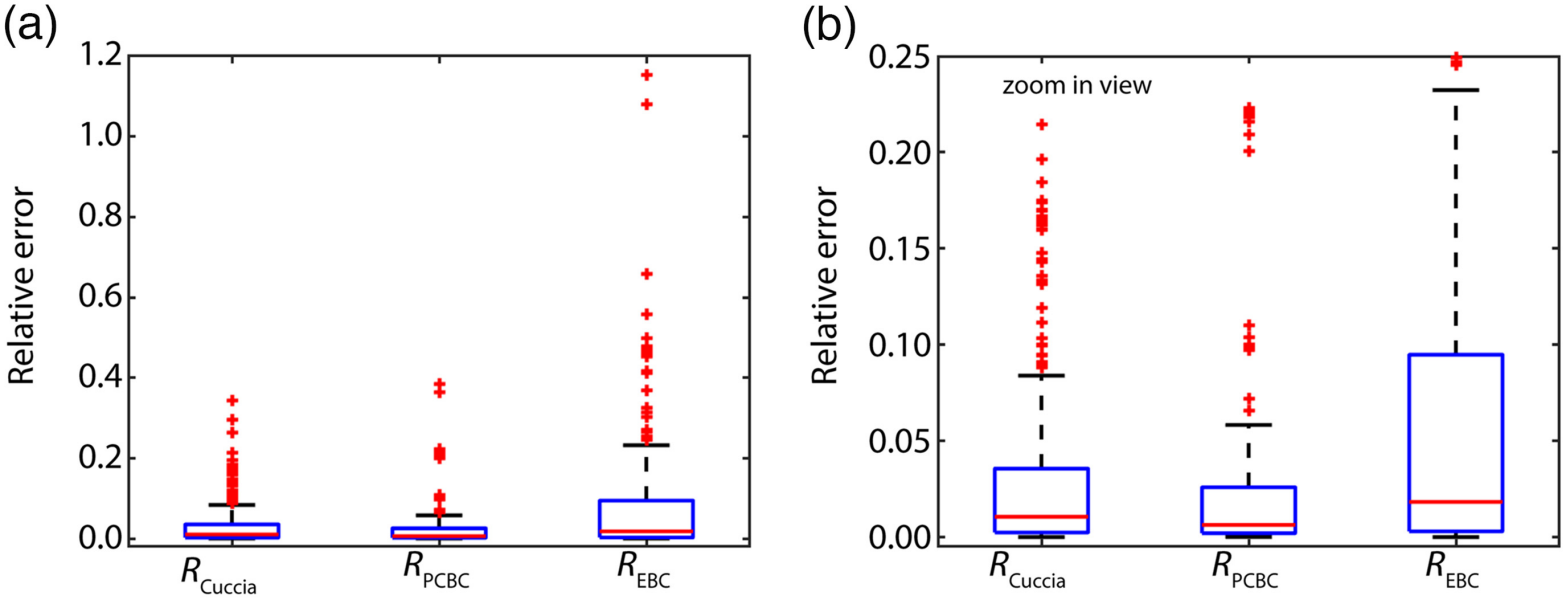
(a) The median and interquartile ranges of the relative error in $R_{\text{Cuccia}}(k)$ [Eq. (4)], $R_{\mathrm{PCBC}}(k)$ [Eq. (10)] and $R_{\mathrm{EBC}}(k)$ [Eq. (11)] versus the simulated reflectance. (b) Zoom-in view of Fig. 3(a). Using $R_{\mathrm{PCBC}}(k)$ instead of $R_{\text{Cuccia}}(k)$ reduces the median relative error in the expected reflectance by 45% from 0.011 to 0.006.

Figure 3 depicts the median and interquartile ranges of the relative error in the reflectance for the three different models compared to all MC simulations. To better show the difference between the results for $R_{\text{Cuccia}}$ and $R_{\mathrm{PCBC}}$ Fig. 3(b) shows a zoom-in view. Using $R_{\mathrm{PCBC}}(k)$ instead of $R_{\text{Cuccia}}(k)$ reduces the median relative error by 45% from 0.011 to 0.006. $R_{\mathrm{EBC}}(k)$ results in a larger median relative error of 0.018, and also a much larger range of errors compared to the other two models.

**Fig. 4:**
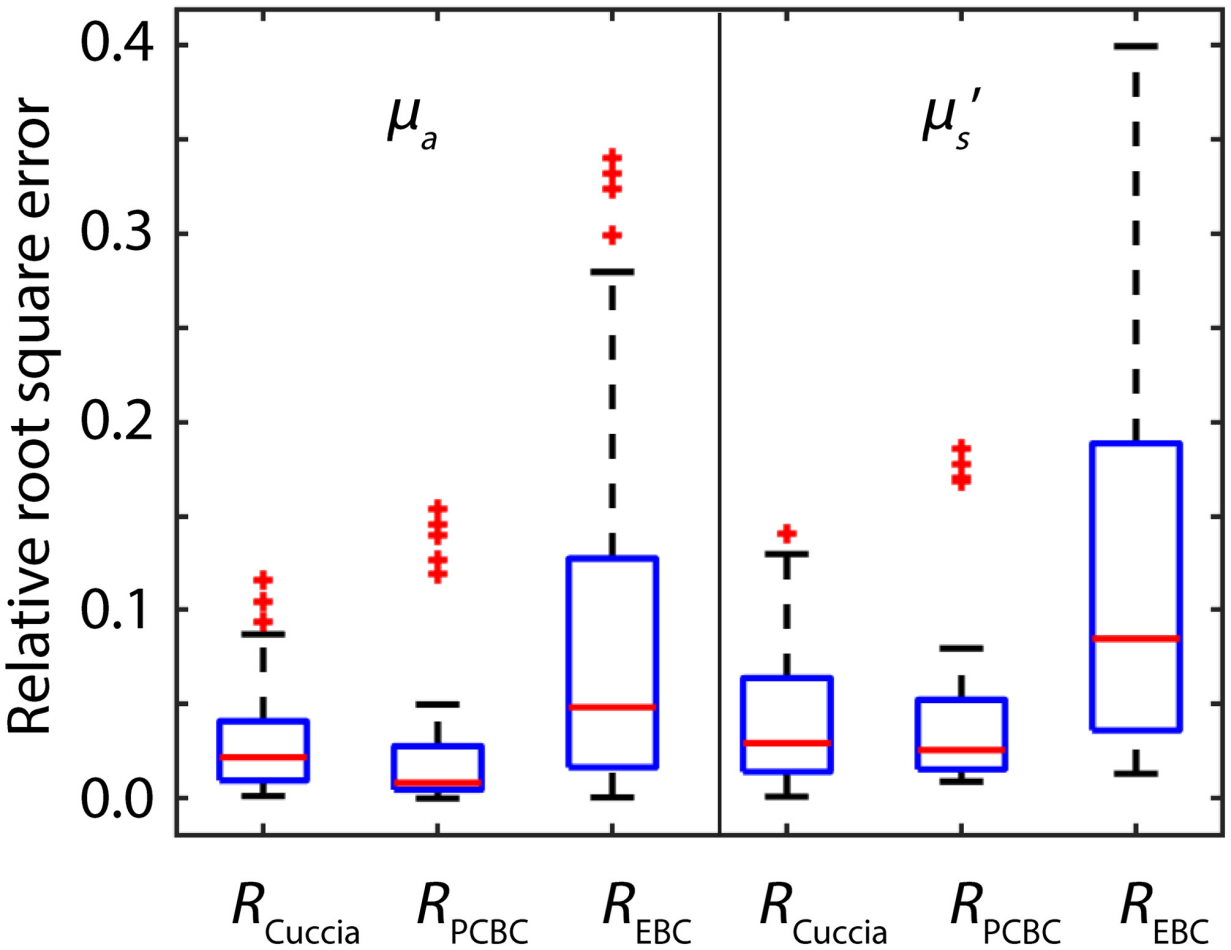
Median and interquartile ranges of the relative error in the extracted values for $\mu_{a}$ and $\mu_{s}^{\prime }$ with $R_{\text{Cuccia}}(k)$ [Eq. (4)], $R_{\mathrm{PCBC}}(k)$ [Eq. (10)], and $R_{\mathrm{EBC}}(k)$ [Eq. (11)]. Using $R_{\mathrm{PCBC}}(k)$ instead of $R_{\text{Cuccia}}(k)$ reduces the median relative error in the extracted value of $\mu_{a}$ by 64% from 0.022 to 0.008 and the median relative error in the extracted value of $\mu_{s}^{\prime }$ by 10% from 0.029 to 0.026.

While Fig. 3 compares the relative errors in the expected reflectance, we also determined the relative errors for each model in extracted values of $\mu_{a}$ and ${\mu_{s}}^{\prime }$ from reflectance values obtained from MC simulations for spatial frequencies of 0 and of $0.5\;{\mathrm{mm}}^{-1}$ (Fig. 4). Using $R_{\mathrm{PCBC}}(k)$ instead of $R_{\text{Cuccia}}(k)$ reduces the median relative error in the extracted value of $\mu_{a}$ by 64% from 0.022 to 0.008 and the median relative error in the extracted value of $\mu_{s}^{\prime }$ by 10% from 0.029 to 0.026. The highest median relative errors were obtained with $R_{\mathrm{EBC}}(k)$: 0.048 for $\mu_{a}$ and 0.085 for ${\mu_{s}}^{\prime }$. Also, the distribution of errors is the largest for $R_{\mathrm{ECB}}(k)$.

## 5 Discussion

We developed a new model for the diffuse reflectance in SFDI that reduces the error in the estimated reflectance and the extracted optical properties compared to the currently used model of Cuccia et al.^2^ Our model was derived by integrating the response function for a pencil illumination R(ρ) over ρ from 0 to ∞ under the PCBC for an extended source (yielding the response for a spatially unmodulated source) and replacing $\mu_{\mathrm{eff}}$ by $\mu_{\mathrm{eff}}^{\prime }=\sqrt{\mu_{\mathrm{eff}}^{2}+k^{2}}$ to take the influence of spatially modulated illumination into account. In other words, we hypothesized and demonstrated the equivalence between (a) computing the Hankel transform of a pencil beam response R(ρ) as in Eq. (5) to (b) direct integration of R(ρ) over ρ from 0 to ∞ followed by the substitution of $\mu_{\mathrm{eff}}$ by $\mu_{\mathrm{eff}}^{\prime }$.

We compared the resulting equations for two models for R(ρ) based on two different boundary conditions, the PCBC and the EBC. We found that the errors in the predicted reflectance, as well as in extracted optical properties were much lower for the PCBC than the EBC. More importantly, for high spatial frequencies measurements are in the subdiffuse regime, where the total reflectance is the sum of a diffuse and a semiballistic component. Thus, for high spatial frequencies, the diffuse reflectance should always be lower than the total reflectance. For the EBC and the model of Cuccia et al., this was not the case. There is no theoretical reason to prefer our approach (starting from a pencil beam illumination) to the approach of Cuccia et al. (starting from a plane wave illumination), or to prefer the PCBC over the EBC as all are based on sound physical principles.^12^ The differences in the reflectance values obtained with the different approaches demonstrate the limitations in modeling diffuse light transport in general. Solving this apparent ambiguity is beyond the scope of this manuscript.

There has been a debate in literature on whether or not the absorption coefficient should be incorporated in the diffusion coefficient for the analytic solutions to the PCBC and EBC.^19^^–^^24^ In this paper, we used $D=1/(3({\mu_{s}}^{\prime }+\mu_{a}))$ to ensure a fair comparison to the model of Cuccia et al., which used absorption in the diffusion coefficient. In the diffuse regime, the reduced scattering coefficient is much larger than the absorption coefficient and the reflectance values obtained with each model would, thus, barely be different regardless of whether or not the absorption coefficient was included in the diffusion coefficient. Therefore, for the purpose of this paper, we could not determine which definition of the diffusion coefficient is more appropriate for SFDI. Even so, the definition of the diffusion coefficient does become important for the development of a subdiffuse model for SFDI. If the subdiffuse reflectance would be modeled as the sum of a diffuse and a semiballistic term,^18^ the accuracy of the diffuse term for high values of $\mu_{a}$ is important. Currently, a model does exist for subdiffuse SFDI,^7^ but it is only valid for one type of tissue phase function and does not include absorption. Therefore, the currently available subdiffuse model cannot be used to interrogate tissue, since there will always be absorbers present and the tissue phase function is generally not known.

Apart from analytical models, approaches exist to extract optical properties from SFDI measurements based on MC simulations—either employing look-up-tables or machine-learning algorithms. MC simulations can include the details of the optical setup that is used (such as angle of incidence and detector numerical aperture) and look-up-tables and machine-learning algorithms can improve the speed of optical property determination. For medical applications where speed is essential, such as endoscopy,^25^ fast algorithms or look-up-tables to extract optical properties are favorable. However, when speed is less important, analytical models provide a few benefits. First, while machine-learning algorithms could overfit the problem and might not provide accurate answers for optical properties that were not simulated, this is much less likely for analytical models. Second, while anybody can use an analytical model since it is a formula that is written out, machine-learning algorithms are often not freely available and can only be reproduced by redoing the MC simulations and retraining the algorithm. Regardless, analytical models are valuable for our fundamental understanding of light transport in general and for SFDI specifically. For example, previously we developed an analytical model for another subdiffuse spectroscopy technique that uses fiber-optic probes: single fiber reflectance (SFR) spectroscopy. We modeled the subdiffuse reflectance as the sum of a diffuse and a semi-ballistic component and identified the new parameter $p_{\mathrm{sb}}$ to incorporate the influence of the phase function on the semi-ballistic component.^26^ Without the analytical model that we developed for the diffuse reflectance in SFR spectroscopy.^27^ we would not have been able to identify the parameter $p_{\mathrm{sb}}$. More importantly, now that we determined that the subdiffuse reflectance in SFR spectroscopy depends on $\mu_{s}^{\prime }$, $\mu_{a}$, and $p_{\mathrm{sb}}$, it is possible to properly perform MC simulations to create look-up-tables or machine-learning algorithms for SFR spectroscopy by including simulations with a range of $\mu_{s}^{\prime }$, $\mu_{a}$ and $p_{\mathrm{sb}}$ values.

In conclusion, we investigated the diffuse reflectance in SFDI by comparing the currently used model of Cuccia et al. to two new models based on integrating the theoretical response function R(ρ) under the EBC and PCBC for a pencil beam illumination over ρ from 0 to ∞ and replacing $\mu_{\mathrm{eff}}$ by ${\mu_{\mathrm{eff}}}^{\prime }$. The model based on the PCBC provides the best results, and reduces the median relative error by 10% for the extracted $\mu_{s}^{\prime }$, 64% for $\mu_{a}$ and 45% for the reflectance. Errors in the expected reflectance can further influence the accuracy of extracted optical properties, since SFDI measurements involve a calibration procedure with a phantom with known optical properties for which the expected reflectance is used to calibrate the setup.

## Code, Data, and Materials Availability

The archived version of the code can be freely accessed and executed through Code Ocean: [https://doi.org/10.24433/CO.6090477.v1]
