# Model for the diffuse reflectance in spatial frequency domain imaging (Erratum)

**DOI:** 10.1117/1.JBO.29.6.069801

**Published:** 2024-06-11

**Authors:** Anouk L. Post, Dirk J. Faber, Ton G. van Leeuwen

**Affiliations:** a The Netherlands Cancer Institute, Department of Surgery, Amsterdam, The Netherlands; b Amsterdam University Medical Centers, Department of Biomedical Engineering and Physics, Amsterdam, The Netherlands

## Abstract

The erratum documents correction of the legend for Fig. 2 in the published article.

This article [*J. Biomed. Opt.* **28**(4), 046002 (2023) doi: 10.1117/1.JBO.28.4.046002] was originally published on 7 April 2023 with an error in the legend of Fig. 2.

Original figure: 

**Figure:**
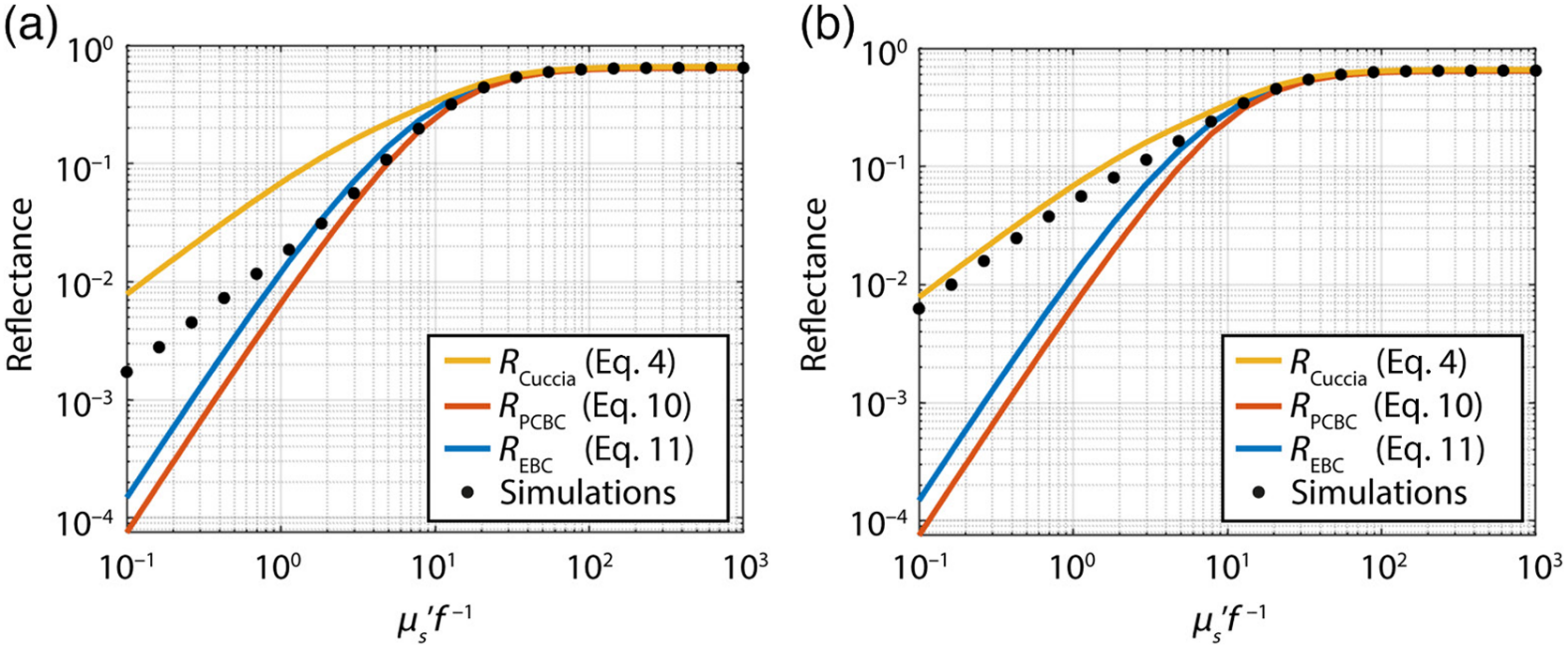


Corrected figure: 

**Figure:**
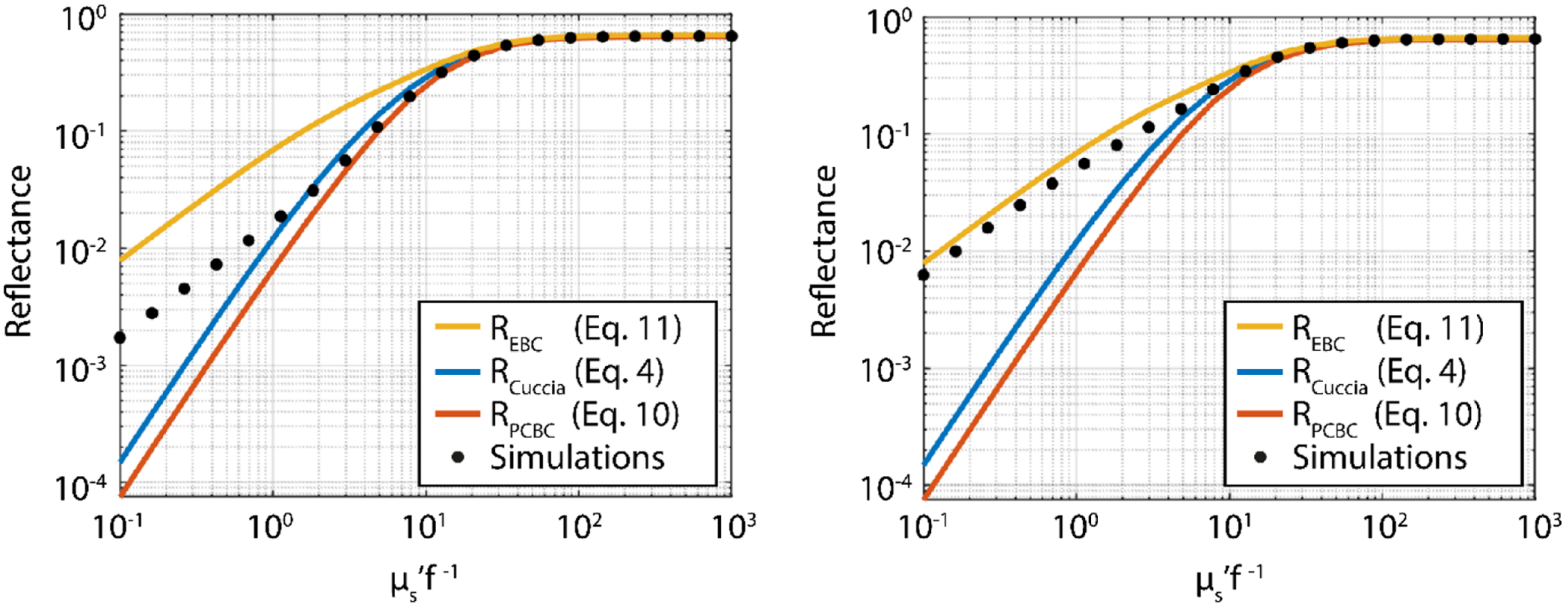


In the original figure, the colored lines in the graph were mislabeled in the legend. The caption for Fig. 2 is unchanged in the corrected version. More importantly, the labeling error impacted neither the analysis nor the description of the results. The article was corrected and republished on 3 June 2024.

